# Rapid, accurate, and reproducible *de novo* prediction of resistance to antituberculars

**DOI:** 10.1128/msphere.00571-25

**Published:** 2025-09-22

**Authors:** Xibei Zhang, Shunzhou Wan, Agastya P. Bhati, Philip W. Fowler, Peter V. Coveney

**Affiliations:** 1 Centre for Computational Science, Department of Chemistry, University College London 4919, London, United Kingdom; 2 Nuffield Department of Medicine, John Radcliffe Hospital, University of Oxford 6396, Oxford, United Kingdom; 3 National Institute of Health Research Oxford Biomedical Research Centre, John Radcliffe Hospital 11269, Oxford, United Kingdom; 4 Advanced Research Computing Centre, University College London 4919, London, United Kingdom; 5 Institute for Informatics, Faculty of Science, University of Amsterdam 1234, Amsterdam, the Netherlands; NC State University, Raleigh, North Carolina, USA

**Keywords:** drug resistance prediction, tuberculosis, computational biology, rifampicin resistance

## Abstract

As one of the deadliest infectious diseases in the world, tuberculosis is responsible for millions of new cases and deaths reported annually. The rise of drug-resistant tuberculosis, particularly resistance to first-line treatments like rifampicin, presents a critical challenge for global health, which complicates the treatment strategies and calls for effective diagnostic and predictive tools. In this study, we apply an ensemble-based molecular dynamics computer simulation method, TIES_PM, to estimate the binding affinity through free energy calculations and predict rifampicin resistance in RNA polymerase. By analyzing 61 mutations, including those in the rifampicin resistance-determining region, TIES_PM produces reliable results in good agreement with clinical reference and identifies abnormal data points indicating alternative mechanisms of resistance. In the future, TIES_PM is capable of identifying and selecting leads with a lower risk of resistance evolution and, for smaller proteins, it may systematically predict antibiotic resistance by analyzing all possible codon permutations. Moreover, its flexibility allows for extending predictions to other first-line drugs and drug-resistant diseases. TIES_PM provides a rapid, accurate, low-cost, and scalable supplement to current diagnostic pipelines, particularly for drug resistance screening in both research and clinical domains.

**IMPORTANCE:**

Antimicrobial resistance (AMR), a global threat, challenges early diagnosis and treatment of tuberculosis (TB). This study employs TIES_PM, a free-energy calculation method, to efficiently predict AMR by quantifying how mutations in bacterial RNA polymerase (RNAP) affect rifampicin (RIF) binding. On simulating 61 clinically observed mutations, the results align with WHO classifications and reveal ambiguous cases, suggesting alternative resistance mechanisms. Each mutation requires ~5 h, offering rapid, cost-effective predictions. An ensemble approach ensures statistical robustness. TIES_PM can be extended to smaller proteins for systematic codon permutation analysis, enabling comprehensive antibiotic resistance prediction, or adapted to identify low-resistance-risk drug leads. It also applies to other TB drugs and resistant pathogens, supporting personalized therapy and global AMR surveillance. This work provides novel tools to refine resistance mutation databases and phenotypic classification standards, enhancing early diagnosis while advancing translational research and infectious disease control.

## INTRODUCTION

Antimicrobial resistance (AMR) ranks among the top 10 global threats to public health and development (1). In 2019, it was estimated that bacterial AMR directly caused 1.27 million (95% UI 0.911–1.71) deaths worldwide and played a contributing role in 4.95 million (3.62–6.57) fatalities (2). Beyond its impact on death and disability, AMR imposes substantial economic burdens. According to World Bank estimates, AMR could lead to an additional US$1 trillion in healthcare costs by 2050, with annual global gross domestic product (GDP) losses ranging from US$1 trillion to US$3.4 trillion by 2030 (3).

AMR arises when bacteria, viruses, fungi, and parasites do not respond to antimicrobial medicines. As a result, it threatens to inhibit many of the advances achieved in modern medicine. Infections like pneumonia, tuberculosis, blood poisoning, gonorrhea, and food-borne diseases are becoming increasingly challenging, and in some cases, they are nearly impossible to treat effectively (4). Of the list, tuberculosis (TB) is a global concern. In 2022, TB caused an estimated 1.3 million deaths, including 167,000 people with HIV. Globally, TB is the second leading infectious killer, only behind COVID-19 and ahead of HIV/AIDS (5).

Caused by the aerophilic intracellular obligate pathogen *Mycobacterium tuberculosis*, TB is an endemic bacterial infection that spreads through airborne transmission, posing a persistent public health challenge worldwide (6). In addition to the lungs, TB can also spread to other organs like the pleura, lymph nodes, and the central nervous system (7).

Drug-resistant tuberculosis (DR-TB) further exacerbates the problem. DR-TB arises from improper use of TB medications, such as incorrect prescriptions, poor-quality drugs, or patients discontinuing treatment prematurely (5). It accounts for 13% of global deaths from AMR, driven by both newly developed resistance and person-to-person transmission, which highlights its severe public health influence (2). Multidrug-resistant tuberculosis (MDR-TB) is a form of DR-TB caused by bacteria that resist at least isoniazid (INH) and rifampicin (RIF), two of the most effective first-line TB drugs. MDR-TB necessitates treatment regimens beyond 18 months and requires second-line drugs, which are often less effective, more toxic, and expensive (8). Additionally, TB caused by bacteria that resist the most effective second-line TB drugs leaves patients with very limited treatment options (9).

TB resistance is elaborated based on genetic mutations in the Mtb genome (*Mycobacterium tuberculosis* genome), which affect drug targets or activate enzymatic functions. Such mechanisms include efflux pumps, drug modification, and impermeability of the cell envelope. Diagnosis of drug-resistant TB and guiding appropriate therapy depend on the detection of such mutations using molecular diagnostics and genome sequencing (6, 10, 11). Current research into drug resistance in TB basically focuses on the elucidation of the molecular mechanisms underlying this phenomenon, the discovery of new drugs, and the repurposing of already existing drugs in the fight against resistant strains. It is also meant to entail the development of quicker and more reliable diagnostic techniques that can identify resistant strains of TB early enough to effectively control the spread of the disease.

Early diagnosis, through universal drug-susceptibility testing and systematic screening of contacts and high-risk groups, is important in effective treatment and management of TB. In 2022, there were 7.5 million new diagnoses of TB, the highest number on record by WHO since the beginning of monitoring in 1995, whereas the estimated cases were 10.6 million (12). The gap between the estimated incidence of TB and diagnoses indicates further work is necessary in improving diagnostic tools. Only 175,650 out of an estimated 410,000 cases (95% UI: 370,000–450,000) of multidrug-resistant TB received treatment, underscoring the diagnostic shortfall even more severely (12).

The main methods for TB diagnosis include microscopy, bacterial culture, and molecular tests (13). Ziehl–Neelsen (ZN) microscopy is the most common technique, especially in resource-limited regions, but it has a sensitivity ranging from 22% to 80%, with the wide variation depending on bacterial load, making it less effective for patients with low bacilli counts (14–16). Bacterial culture methods like Lowenstein–Jensen are highly specific and sensitive, with sensitivity between 80% and 85%, but they take 4–6 weeks to provide results (17), which conflicts with the requirement of early diagnosis. Among molecular tests, GeneXpert MTB/RIF has a sensitivity of 89% and specificity of 99%, and is a fast TB/DR-TB test (in less than 2 h) (18, 19). However, it is expensive and is also unable to distinguish between viable and non-viable bacteria. These limitations call for further research on the molecular mechanism of resistance to anti-TB drugs and the development of diagnostic technology.

Limitations of traditional diagnostic methods, particularly in AMR, call for more sensitive approaches. More complex forms of resistance, particularly in diseases like TB, need advanced diagnostic functionalities and more specific information on the molecular aspects of resistance mechanisms. It is in this area that computational methods may be used for significant advancement in AMR studies.

In the study of AMR, computational methods have made significant progress thanks to advances in molecular biology, bioinformatics, cheminformatics, as well as the increasing power of high-performance computing (HPC). Going beyond the initial stage of the interactions between proteins and ligands by using molecular docking, the method has been extended toward molecular dynamics (MD) simulations, capable of modeling the resistance mechanism that occurs in such complex molecular systems as the TB-related proteins. Development milestones concerning the formulation of the physics-based (PB) computational methods represent, at first, the early introduction of molecular docking in the 1980s for the prediction of drug binding (20), followed by MD simulations in the 1990s studying molecular time-dependent behavior (21). In the 2000s, free energy calculation methods were introduced for more precise prediction of binding affinities (22, 23). Very recently, there have been integrations of HPC with these methods, enabling large-scale simulations for the generation of more accurate predictions, especially using ensemble-based methods (24). Tools like AutoDock, Glide, GROMACS, and AMBER have been widely used for drug discovery and molecular simulations, whereas recent advancements in machine learning (ML) also help enable the prediction of resistance mutations using large data sets (25). Recent efforts have prospectively evaluated resistance mutations using blinded data, moving computational predictions closer to clinical application (26, 27).

The widespread availability of high-throughput technologies provides current opportunities for ML and big data to improve predictions and uncover new mechanisms of resistance that will accelerate drug discovery. However, several challenges remain, including the need for high-quality experimental data, substantial computational resources, and the complexity inherent in the mechanisms of drug resistance, where integration of biological insight with computational models is essential.

Binding affinity provides a computational route to clinical decision support. Mutations often lead to drug resistance by reducing the binding affinity between an antibiotic and its target. To quantify this, we can calculate the difference in binding free energy (ΔΔG) between the wild type and mutant proteins, using relative binding free energy (RBFE) methods. The most frequently used RBFE method is an alchemical approach, where the wild-type amino acid is gradually and unphysically changed into the mutant in a simulation, with energy differences measured at each step. These calculations, in turn, inform how the mutation impacts drug binding, thereby predicting the possible drug resistance.

RBFE has seen widespread application in drug-lead optimization to predict the impact of small changes in a drug on its binding to a target, reaching an accuracy around 1 kcal/mol (28–30). Studies have shown RBFE to be effective in predicting resistance mutations in various diseases (31–35), including those related to drug resistance in small proteins, such as those involved in trimethoprim resistance in *Staphylococcus aureus* and *Haemophilus* (32, 36, 37).

Although RBFE has been highly effective in predicting drug resistance in small protein systems (27, 38–40), applying it to larger macromolecular complexes remains challenging due to accuracy and computational limitations. However, the increasing availability of detailed 3D models of macromolecular structures, alongside advances in HPC, has made drug-resistance prediction more promising, as the work reported in this paper attests.

By making the most of MD simulations combined with relative binding free energy (RBFE) calculations, we are able to predict RIF resistance mutations in the RNA polymerase (RNAP) protein, which is encoded by the rpoB gene, with high accuracy; RIF acts by binding to the β-subunit of the RNAP, preventing RNA extension (Fig. 3 shows their positions).

In this study, we apply an ensemble-based method called TIES_PM (Thermodynamic Integration with Enhanced Sampling for Protein Mutations) (41, 42) to predict drug resistance in the RNAP-RIF system. The innovation of this work lies not in the computational framework *per se*, but in its large-scale application (RNAP-RIF system includes over 440,000 atoms), the integration with curated phenotypic data, and the systematic evaluation across 61 clinically observed mutations. The paper is structured as follows: in section 2, we introduce the method in detail. In Section 3, we present the prediction results of drug resistance, including a number of mutations falling within or outside the rifampicin resistance-determining region (RRDR). In section 4, we discuss the results from section 3, highlighting the broader implications of this method for potential biomedical and clinical applications.

## MATERIALS AND METHODS

This section introduces the underlying theory of free energy calculation, the core computational method (TIES_PM), the protein and ligand preparation, the simulation process, and the post-processing of data generated by the simulations.

### Free energy theory and TIES_PM

The binding affinity between a drug and its target protein is an important property in both drug design and drug resistance prediction, indicating the ability of the drug to interact with the target protein. The major idea is that ligand binding is driven by changes in Gibbs free energy ∆G. The more negative ∆G is, the more stable the complex of protein and drug will be, indicating stronger binding. Therefore, the change in Gibbs free energy is calculated as


(1) $$\Delta G_{binding}^{\;}=\left(G_{A}-G_{B}\right)$$

Where $\Delta G_{binding}$ is the change in Gibbs free energy, $G_{A}$ is Gibbs free energy of the complex, and $G_{B}$ is the summation of Gibbs free energies of protein and drug.

For drug resistance prediction, if ∆G becomes less negative after the mutation occurs, indicating a lower affinity between the drug and the mutated targeted protein, it suggests the occurrence of drug resistance. Conversely, a decrease in free energy (i.e., a more negative ΔG) corresponds to increased binding affinity, indicating that the mutation remains susceptible.

TIES_PM (Thermodynamic Integration with Enhanced Sampling for Protein Mutations) is an open-source computational methodology designed to compute the changes in binding free energies due to protein mutations (34, 41, 42). This extended version of the standard TIES approach incorporates protein mutations within the simulation with a single inhibitor (34, 41, 43), enabling detailed analysis of how particular amino acid substitutions alter the characteristics of protein-ligand interactions.

**Fig 1:**
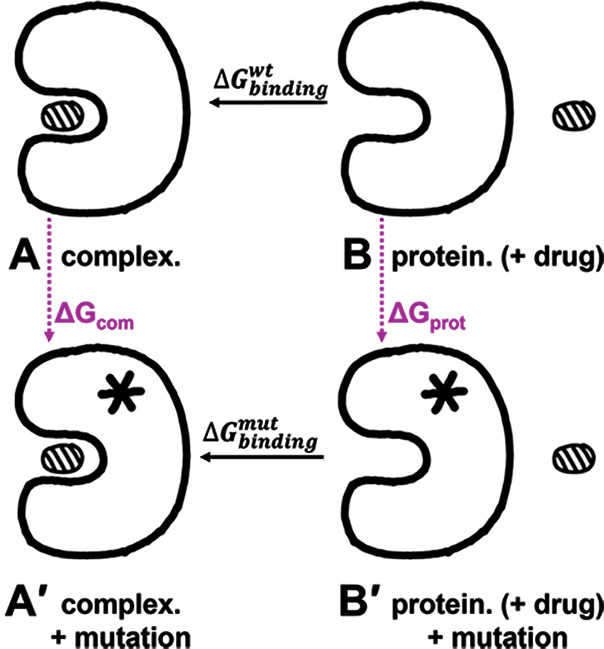
Changes in Gibbs free energy (ΔΔG) in protein-drug binding with and without mutation. State **A** represents the bound complex (protein + drug), and state **B** represents the unbound state (protein + drug). The presence of a mutation introduces states A′ (mutant complex) and B′ (mutant protein + drug). $\Delta G_{com}^{\;}$ and $\Delta G_{prot}^{\;}$ denote the changes in free energy for the respective states, whereas $\Delta G_{binding}^{wt}$ and $\Delta G_{binding}^{mut}$ reflect the binding free energy differences before and after mutation.


The basic idea of TIES_PM is to save computational resources by altering the problem (34). Originally, we wanted to compare the binding affinities before and after the mutation. Therefore, a direct solution is to calculate the difference between the changes in Gibbs free energy of the original protein-ligand pair $\Delta G_{binding}^{wt}$ and the mutated protein-ligand pair $\Delta G_{binding}^{mut}\;$ (Fig. 1). However, as we decompose each energy term and make equivalent transformations, we can alter formula (1) to (2), where the difference between the two protein-ligand pairs (B-A and B′-A′ in Fig. 1) changes into the difference between processes A-A′ and B-B′. The latter are called alchemical processes, since these intermediate steps are unphysical, although the end states are real ones—the difference between the end states gives the ΔΔG since free energy is path-independent.

(2) $$\begin{array}{rl} \Delta \Delta G & =\Delta G_{binding}^{mut}-\Delta G_{binding}^{wt}\\ & =\left(G_{A^{\prime }}-G_{B^{\prime }}\right)-\left(G_{A}-G_{B}\right)\\ & =\left(G_{A^{\prime }}-G_{A}\right)-\left(G_{B^{\prime }}-G_{B}\right) \end{array}$$

(3) $$\Delta \Delta G=\Delta G_{com}-\Delta G_{prot}\;\;$$

When ΔΔG<0, mutated protein exhibits better affinity, meaning susceptible; instead, when ΔΔG >0, the original protein exhibits better affinity, meaning it is resistant. Because the change in the system is much smaller for the alchemical process compared with the change in protein-ligand pairs, the relative binding free energy (RBFE) calculation will reduce the sampling space, save the calculation resources, and increase the accuracy of the calculations.

**Fig 2:**
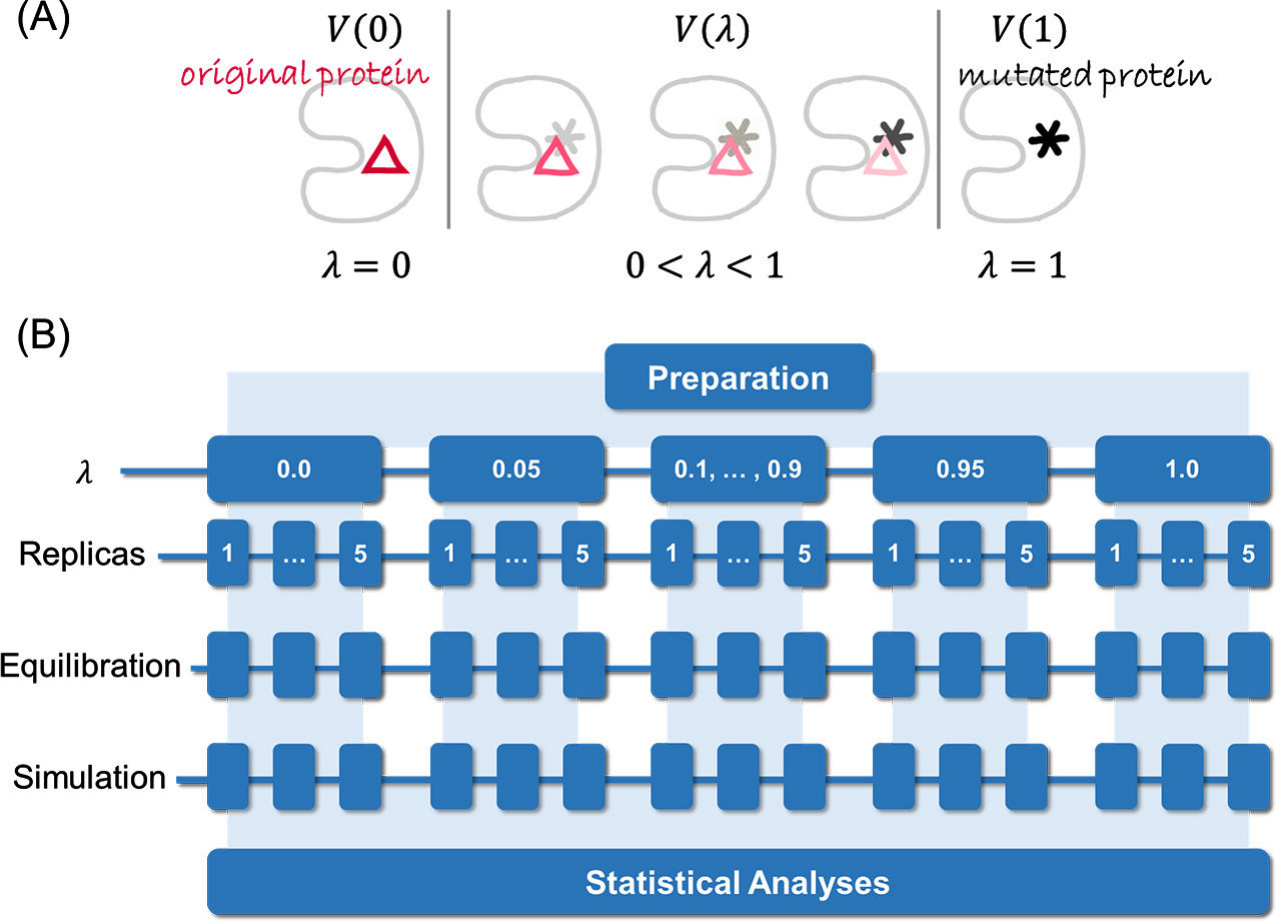
The alchemical transformation process and ensemble simulation setup. Figure (**A**) illustrates the relationship between the degree of the mutation parameter λ and the alchemical mixture states. 0 < λ < 1 here are the intermediate transformation states, and although three states are shown here, in practice, this interval includes a number of states. Here, in the current work, eleven intermediate λ states are utilized along with the two ends (λ = 0 and λ = 1), giving a total of 13 states. Figure (**B**) shows the ensemble simulation architecture, where five replicas are employed for every state (41).

To alchemically mutate a protein from wild-type to a mutant state at an atomic level, some intermediate states, or windows, are defined between the two end points (wild-type and mutant) in TIES_PM, as shown in Fig. 2A. Each of them must be simulated during the computation of the free energy differences. In our work, 13 windows are set, each being a mixture of the two physical states in different portions under control by the degree of the mutation parameter λ. The wild and the mutant protein are represented by λ=0 and λ=1 states, respectively, and the intermediate mixed states are represented by $\lambda \in \left(0,1\right)$.

The change in the Gibbs free energy is determined using thermodynamic integration (TI), based on the following equation 4; (41):


(4) $$\Delta G=\int_{0}^{1}\frac{\partial G\left(\lambda \right)}{\partial \lambda }d\lambda =\int_{0}^{1}{\left\langle \frac{\partial V\left(\lambda ,x\right)}{\partial \lambda }\right\rangle }_{\lambda }d\lambda$$

Where λ is the degree of the mutation, $V\left(\lambda ,x\right)$ is the potential energy, and ${\left\langle \frac{\partial V\left(\lambda ,x\right)}{\partial \lambda }\right\rangle }_{\lambda }$ is an ensemble average of the potential energy derivative in state λ.

Simulations are carried out for five replicas at each of these windows so that a wide range of conformations can be sampled. This gives an ensemble (Fig. 2B) where the prediction gets more precise by capturing conformational diversity often missed by one-off simulations. This ensemble approach is essential because molecular dynamics is inherently chaotic—single simulations cannot reliably capture this diversity or ensure reproducibility. By using multiple replicas, we control errors and improve prediction accuracy efficiently (44). TIES_PM will quantify exactly how these kinds of mutations impact drug binding and activation, by computing the free energy differences between ligand-bound and apo-protein states. The mean and standard error (SE) were estimated using bootstrap (45) resampling (B = 10000), whereas the 95% confidence intervals were calculated from Student’s t-distribution (46), as $\overset{-}{X}\pm t∙SE$ with $t_{0.975,\;4}=2.776$ for *n* = 5 replicas (i.e., degrees of freedom = 4). This approach aligns with the classical TIES framework, which has been thoroughly benchmarked across multiple targets, including TYK2, demonstrating robust accuracy (41, 47–49). TIES-PM has been successfully applied to protein mutations in diverse systems such as FGFR3 kinase (34) and estrogenic receptor (37), where convergence analysis confirms that 4 ns production runs are sufficient for most cases.

### System preparation and simulation

#### Protein and ligand preparation

Preparing computing models of proteins and mutations requires a number of steps.

First, we used the crystal structure of the Mycobacterium tuberculosis RNA polymerase holoenzyme in complex with rifampicin (PDB ID: 5UH6) as the starting model. As is common with crystallographic structures, the model can contain missing loops or atoms when retrieved from a crystal structure in a PDB file. In order to complete the missing part, structures predicted using AlphaFold2 (50) are used to complete the missing segments, following alignment with the native X-ray structure based on common, selected regions.

Second, redundant conformers are removed from proteins with multiple conformers in order to maintain uniformity in the structure.

Next, “reduce” from AmberTools (51) is run to add proper protonation states of the amino acids so that the biologically correct state of the protein is correctly represented. In the case of a drug molecule like RIF, bond correction and hydrogens are added in order to be ready for MD simulations. Then, parameterization is performed based on standard force fields (52–55), such as ff14SB (56) for protein, and GAFF2 (57) for the drug molecule. A dual topology RBFE is used to introduce mutations. Both wild-type and mutant side chains coexist, and their interactions with the environment are scaled accordingly to reproduce the gradual transition between the wild-type and the mutant state. The system is then set up by solvating the structure, adding ions, and applying periodic boundary conditions, with parameters like the size of the simulation box, buffer distance, ionic bonds, and number of atoms being controlled. All this is done using Amber’s tleap tool in order to prepare the necessary input files. The system is then thoroughly checked for correctness before submitting it to a supercomputer to run simulations.

#### Simulation process

Energy minimization and 1 ns equilibration are conducted before production runs, which involve MD simulations of 4 ns for each replica and five replicas in the ensemble using NAMD (58). Trajectories and energy derivatives are saved every 10 ps for further analysis. The simulations are executed on the Argonne National Laboratory’s Polaris supercomputer, equipped with AMD EPYC processors and NVIDIA A100 GPUs and a peak capacity of 44 petaflops (59). The simulation rate of our systems is around 24 ns/day, the wall clock time is 1.3 h for the equilibration phase and 4.1 h for the simulation phase for one replica.

Additionally, TIES_PM simulations are compared with clinical data to validate the predictions. For instance, data from the WHO resistance catalog (60–63), available as a CSV file, includes classifications of mutations and some associated clinical evidence in the EVIDENCE column formatted as JSON. These data are informative in terms of the clinical significance of mutations and help classify mutations as resistant (R) or susceptible (S) to best represent clinical realities, especially given the lack of experimental ΔΔG values for RNAP-RIF mutations. Including this evidence is important to ensure that TIES_PM simulations best represent clinical realities, even with acknowledgment that quantitative values, for example, minimum inhibitory concentrations or titration calorimetry, are optimally suited for drug resistance and susceptibility determination.

#### RRDR and the choice of mutations

The rifampicin resistance-determining region (RRDR) (60, 64–66) in *Mycobacterium tuberculosis* typically spans amino acids 428–452 of the rpoB gene, where most clinically observed rifampicin (RIF) resistance mutations occur. These residues form part of the drug’s binding pocket on the β-subunit of RNA polymerase (RNAP); hence, mutations here often disrupt RIF’s inhibitory action.

**Fig 3:**
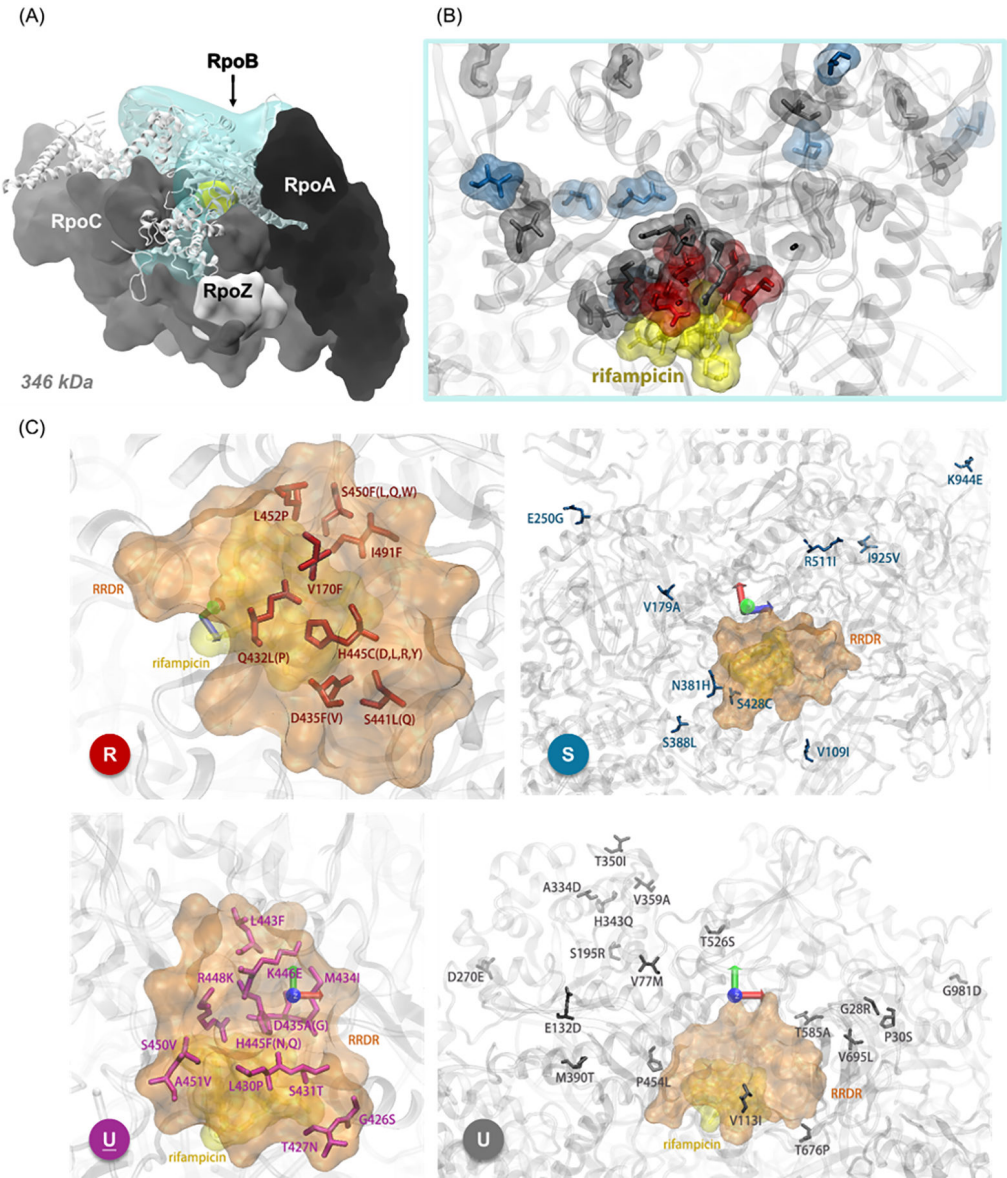
*M. tuberculosis* RNA polymerase (RNAP) structures. (**A**) shows the relative position of rpoB (cyan) in the protein, and all RNAP subunits are shown in surface view, with nucleic acids omitted in close-ups; (**B**) shows the mutations within rpoB, depicted as associated with antibiotic resistance (red), susceptibility (blue), and unknown type (gray), positioned relative to antibiotic binding sites (yellow); and (**C**) shows the spatial distribution of mutations relative to RRDR region and the drug-binding site, grouped by clinical phenotypes: R (resistant), S (susceptible), U (unknown within RRDR), and U (unknown outside RRDR).

However, not all non-synonymous mutations in the RRDR confer resistance (e.g., rpoB L443F [64]); some resistance-conferring mutations (e.g., rpoB I491F, V170F) lie outside it. Thus, although the RRDR is a critical target for RIF resistance detection, more comprehensive mutation screening remains important. In our study, we selected 61 mutations, with roughly half inside and half outside the RRDR (Fig. 3).

### Resistance classification and data validation

#### Clinical threshold

A positive change in binding free energy (ΔΔG > 0) means that the antibiotic binds less effectively to its target after a mutation, thus indicating drug resistance. Clinically, a sample is deemed “resistant” if its minimum inhibitory concentration (MIC) exceeds a critical value, generally the epidemiological cut-off value (ECOFF/ECV)—the 99th percentile MIC of wild-type samples. Following a thermodynamic framework adapted from enzyme inhibition kinetics, MIC distributions of resistance mutations can be translated into a ΔΔG threshold (32). For the RNAP–RIF system, this yields an expected ΔΔG of ~1.2 kcal/mol, based on ECOFF/ECV values from the CRyPTIC Consortium (62) and geometric mean MICs of resistant variants (31, 32).

#### Classification of SOLOs and phenotype ratio

A key concept is the definition of mutations as SOLO (single-occurrence, lone-observation) events—instances where a mutation occurs exclusively without co-occurring variants in a sample. By comparing the frequency of R/S within SOLO mutations across samples, their resistance effect classification is assigned. The C138R mutation has, for instance, always yielded resistance and can thus be reliably categorized as R, whereas mutations like I133T produce variable R/S outcomes, and hence, their resistance classification is unknown.

To better describe the original structure of the reference clinical data, we define the “phenotype ratio” as R or S frequency over total SOLOs. This provides a basis on which to find the clinical importance of a mutation by comparing R/S results across samples. Some mutations, such as D435G, have SOLOs with 50% R and 50% S distribution yet are resistant, possibly because they are within the RRDR and do not have definitive phenotypic data. Clinical data thus need to be critically interpreted based on their data structure.

#### Statistical assessment of consistency between data sets

To make a comparison of our computations with those in previous studies, we used the paired *t*-test and F-test. The *t*-test finds mean differences, and the F-test finds variances (67). We also tested model performance in sensitivity (true positive rate, proportion of resistant mutations correctly predicted) and specificity (true negative rate, proportion of susceptible mutations correctly predicted) (68). Additionally, the F1 score (69) (harmonic mean of precision and recall) was used to evaluate how well the model balances correctly detecting resistant mutations while avoiding misclassifying susceptible ones.

## RESULTS

In this section, we first performed calculations on seven important mutations. Three of them are clinically resistant, whereas four are susceptible. We have also compared the results with those from the previous study by Fowler et al. (31). With the confidence built from these predictions, 61 mutations are calculated altogether, increasing the study’s convincingness with greater findings from the results.

### Comparison with the previous computational study

**Fig 4:**
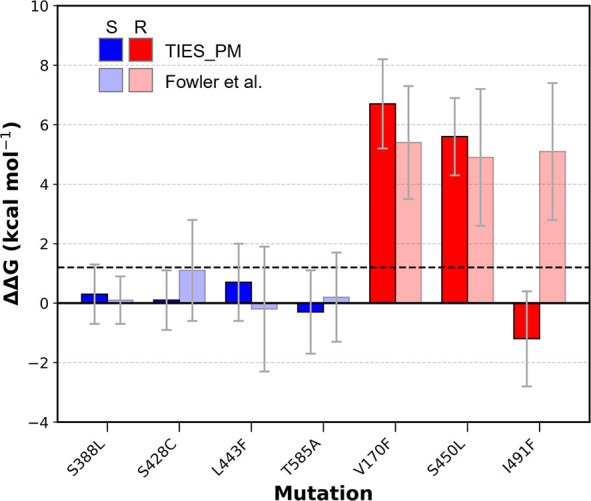
Our results align well with both clinical data and the previous study (31). The impact of the listed mutations on the free energy of rifampicin binding to RNAP is presented. We display the deep-colored bars exhibiting our findings, and the light-colored bars exhibit those of the previous investigation (31). Susceptible (S) mutations, as revealed by clinical screening, are colored blue, whereas resistant (R) mutations are in red. The dashed line marks the ΔΔG threshold corresponding to the epidemiological cutoff for rifampicin resistance; mutations with values above this threshold are considered resistant in *M. tuberculosis*. Error bars represent 95% confidence intervals.

The first set of seven mutations was selected based on their positions and effects on rifampicin resistance. S450L is located within the RRDR of rpoB. V170F and I491F, while lying outside the RRDR, are near both the S450L mutation and the antibiotic binding site for conferring resistance; I491F is typified by having variable MICs. The negative controls are L443F, which is inside the RRDR and near the binding site but does not generate resistance, S388L, and T585A, which are further away from the binding site and arise in clinical specimens without conferring resistance. S428C, an RRDR mutation with no resistance association and with minimal effect on the sidechain, was chosen as an artificial negative control since it is not observed in clinical samples and is oriented away from the drug in its sidechain. The prediction results were compared against the previous work (31), as shown in Fig. 4 and Table 1.

**TABLE 1:** The calculated impact of the listed mutations on rifampicin’s binding free energy to RNAP is presented^*a*^

Mutation	ΔΔG	Error	R/S	ΔΔG'	Error'	R/S'	Exp.	Phenotype ratio	Sample size
S388L	0.3	1.0	U	0.1	0.8	S	S	100.0%	1
S428C	0.1	1.0	S	1.1	1.7	U	S	–	–
L443F	0.7	1.3	U	−0.2	2.1	U	S	100.0%	1
T585A	−0.1	1.3	S	0.2	1.5	U	S	–	–
V170F	6.7	1.5	R	5.4	1.9	R	R	97.6%	41
S450L	5.6	1.3	R	4.9	2.3	R	R	97.9%	8912
I491F	−1.6	1.8	S	5.1	2.3	R	R	55.5%	173

^*a*^ ΔΔG, Error, and R/S reflect calculations from our TIES_PM study, while values marked with a prime (′) (ΔΔG′, Error′, R/S′) correspond to results from the previous study (31). Exp., phenotype ratio, and sample size represent the resistance classification, clinical phenotype ratio, and sample size of the clinical data, respectively. All energy values from calculations are in kcal/mol. The symbol “–” suggests insufficient data available to calculate the phenotype ratio.

All four negative controls (S388L, S428C, L443F, and T585A) were predicted to have no effect on the drug’s effectiveness against RIF. Both resistance-conferring mutations (V170F and S450L) show not only positive ΔΔG values but also exceed the ECOFF/ECV-derived clinical threshold, confirming the fact that they confer resistance. The disputed mutation I491F was predicted to be susceptible, contrary to the clinical experimental results (as shown in Table 1). However, it is important to note that the clinical assessment of I491F has a phenotype ratio of only 55.5%. The reliability of clinical data significantly impacts the assessment of prediction accuracy, as demonstrated in the following section.

In addition, the paired *t*-test yielded t = −0.672 with a *P*-value of 0.527, indicating no significant difference in the mean ΔΔG values between our results and the previous study (31). Similarly, the F-test produced F = 0.204 with a *P*-value of 0.660, suggesting that the variance of the two data sets is also statistically similar. These results confirm that our computed values are consistent with the previous study, supporting the robustness of the TIES_PM approach.

Notably, our predictions exhibit a higher level of classification confidence, as the deviations that cross the resistance threshold are fewer in number and smaller in magnitude compared to the previous study (31). This indicates that TIES_PM provides a more refined and reliable assessment of resistance and susceptibility, reducing ambiguity in borderline cases.

### Predictions among the most common mutations

To comprehensively assess our TIES_PM method, we predicted drug resistance for a total of 61 mutations, integrating those with established clinical classifications as well as those initially labeled as unknown type (60). The mutations were selected to encompass key rifampicin resistance-associated residues, covering diverse phenotypic categories (resistant, susceptible, unclassified), thermodynamic stability ranges (ΔΔG: −1.6 to 18.2 kcal mol⁻¹), and clinical phenotype ratios (0%–100%), thereby rigorously evaluating the TIES_PM method’s robustness across mechanistically ambiguous and statistically uncertain scenarios.

**Fig 5:**
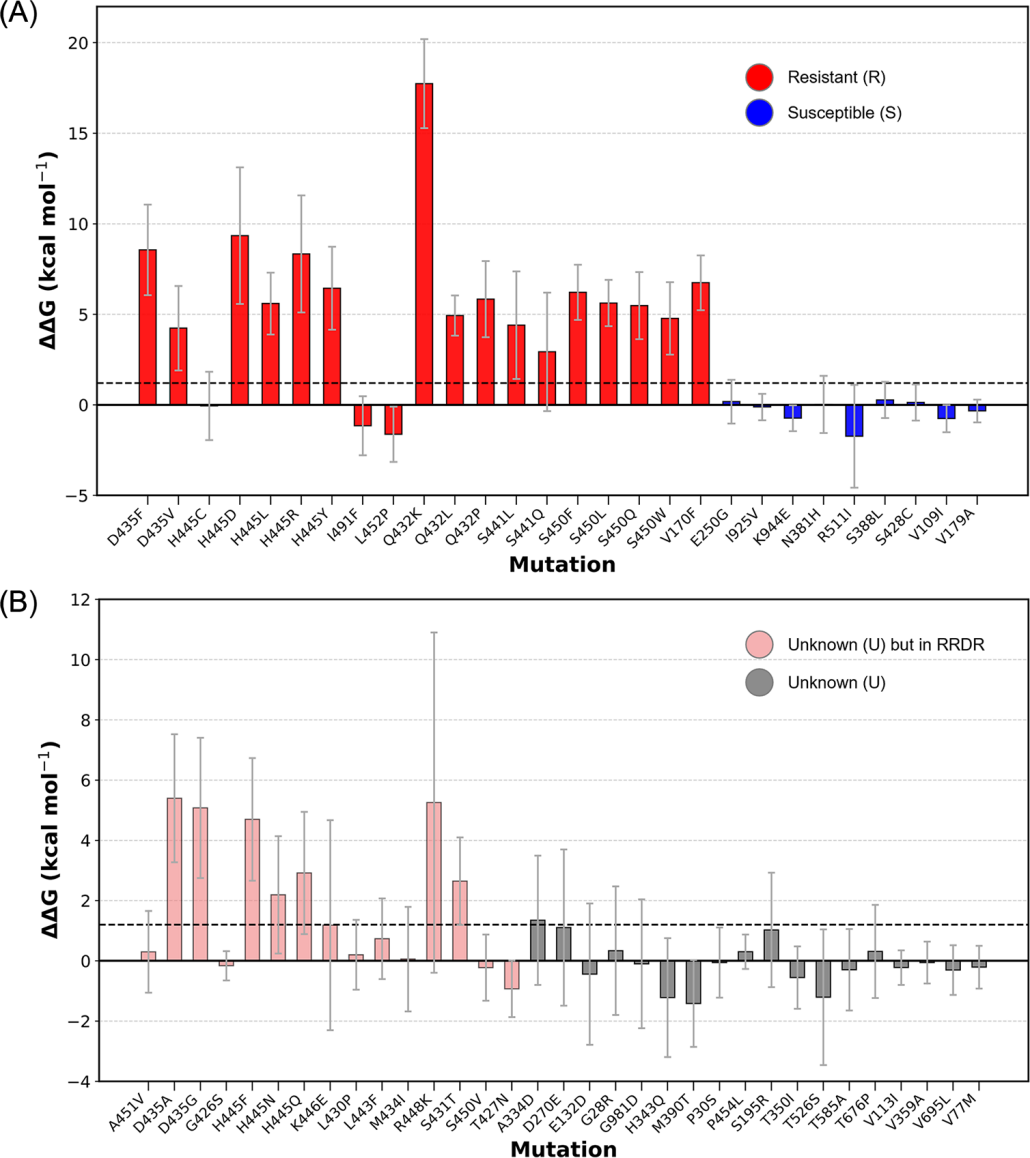
The calculated impact of 61 mutations on rifampicin’s binding free energy to RNAP. Mutations are categorized into two groups based on WHO clinical classifications: (**A**) mutations classified as resistant or susceptible in the WHO data set, represented in red and blue, respectively; (**B**) mutations initially classified as unknown due to insufficient or conflicting clinical evidence. In (**B**), all mutations are classified as unknown, but those occurring in the RRDR region are shown in light red and conservatively classified as resistant by WHO for safety considerations. Dashed lines mark the ΔΔG value corresponding to the epidemiological cutoff for rifampicin; values above this indicate clinical resistance in *M. tuberculosis*. Bars represent the mean ΔΔG for each mutation compared to the wild type, with 95% confidence intervals displayed.

As shown in Fig. 5, we classified the mutations into two groups: group A includes mutations for which the WHO has provided a definitive classification as resistant (R) or susceptible (S), whereas group B consists of mutations originally classified as unknown due to insufficient or conflicting clinical evidence. This distinction allows us to evaluate our approach across both well-characterized and ambiguous cases. Detailed numerical values are provided in Table S1 from the https://github.com/UCL-CCS/TIES_PM_RNAP_RIF .

From Fig. 5A, nineteen mutations are classified as R, and nine as S. Among these, our model correctly predicted 15 resistant mutations and all nine susceptible mutations, even considering error bars. The sensitivity and specificity are 78.95% and 100%, respectively, whereas the F1 score reached 88.24%, reflecting a strong balance in performance. These results demonstrate the reliability of our model in accurately identifying resistant mutations while minimizing false positives, making it a robust tool for drug resistance prediction.

Among the four resistant mutations (H445C, I491F, L452P, and S441Q) that were misclassified or inaccurately predicted, two exhibit low clinical phenotype ratio: I491F, previously discussed in the preceding section, was found resistant in 96 out of 173 SOLO tests, yielding a phenotype ratio of only 55.5%; similarly, L452P was clinically identified as resistant in 168 out of 259 SOLO tests, with a phenotype ratio of only 64.9%, as listed in Table S1, https://github.com/UCL-CCS/TIES_PM_RNAP_RIF. In contrast, H445C and S441Q exhibit normal phenotype ratios above 90%. The average ΔΔG for S441Q exceeds the decision threshold, but its error margin is relatively large. Increasing the number of replicas is expected to improve accuracy, thereby enabling the correct prediction of resistance. As for H445C, possible explanations for the discrepancy will be further discussed in the discussion section.

Comparing Fig. 5A and B, a significant increase in error bars is observed in Fig. 5B, which corresponds to mutations initially categorized as unknown by WHO. This classification arises from insufficient or conflicting clinical data, and the larger error bars in our calculations further reflect the complexity of these mutations. Accordingly, for predictions where the error bars span both sides of the resistance threshold, we consider them as “unknown type” with a label “U.” This is not unusual, especially when the estimated values are close to zero. This is indeed one reason some mutations are classified as “Unknown.” Their impact on drug susceptibility is minimal, leading to clinical observations that may go either way. Thus, in terms of uncertainty as indicated by computational errors, a consistency exists between our model’s predictions and the classification challenges inherent in these mutations. To investigate this further, we assessed λ-point overlap, inter-replica consistency, and ΔΔG convergence (see Supplementary Information S3–S5, https://github.com/UCL-CCS/TIES_PM_RNAP_RIF). Most systems show stable free energy estimates and sufficient sampling, in line with the standards proposed by Zhang et al. (40). These findings confirm that the observed uncertainties are not due to sampling deficiencies but reflect the intrinsic structural or physicochemical complexity of the mutations, such as charge changes, bulky side chains, or local rearrangements.

Furthermore, mutations classified as resistant based on their location—highlighted in light red in Fig. 5B—exhibit a higher mean ΔΔG despite larger errors, aligning with their predicted resistance tendency.

Overall, these results support the reliability of the TIES_PM method. Our prediction is statistically consistent with the previous study (31) as confirmed by a paired *t*-test (t = –0.672, *P* = 0.527) and an F-test (F = 0.204, *P* = 0.660). Building on this validation, our TIES_PM model demonstrated robust predictive performance with a sensitivity of 78.95%, specificity of 100%, and an F1 score of 88.24%, reliably distinguishing resistant from susceptible mutations.

## DISCUSSION

Our predictions of RIF resistance in *M. tuberculosis* using the TIES_PM method demonstrated strong accuracy and agreement with clinical reference data. When compared with the previous study (31), our TIES_PM method yielded more precise results with fewer unknown phenotypes. This can be attributed to it using more replicas and a longer simulation time. Where we ran five replicas with a 4 ns simulation time, their method employed three independent 0.5 ns simulations per mutation. This improves the stability and reliability of the free energy calculations.

Among all the 61 mutations, our TIES_PM predictions achieved high specificity (100%), sensitivity (78.95%), and F1-score (88.24%) for WHO-classified R/S mutations, further confirming the model’s reliability. As presented in the results section, among the nineteen mutations classified as R by WHO clinical testing data, three were predicted as S or unknown by the TIES_MD calculations. These cases will be discussed next.

**Fig 6:**
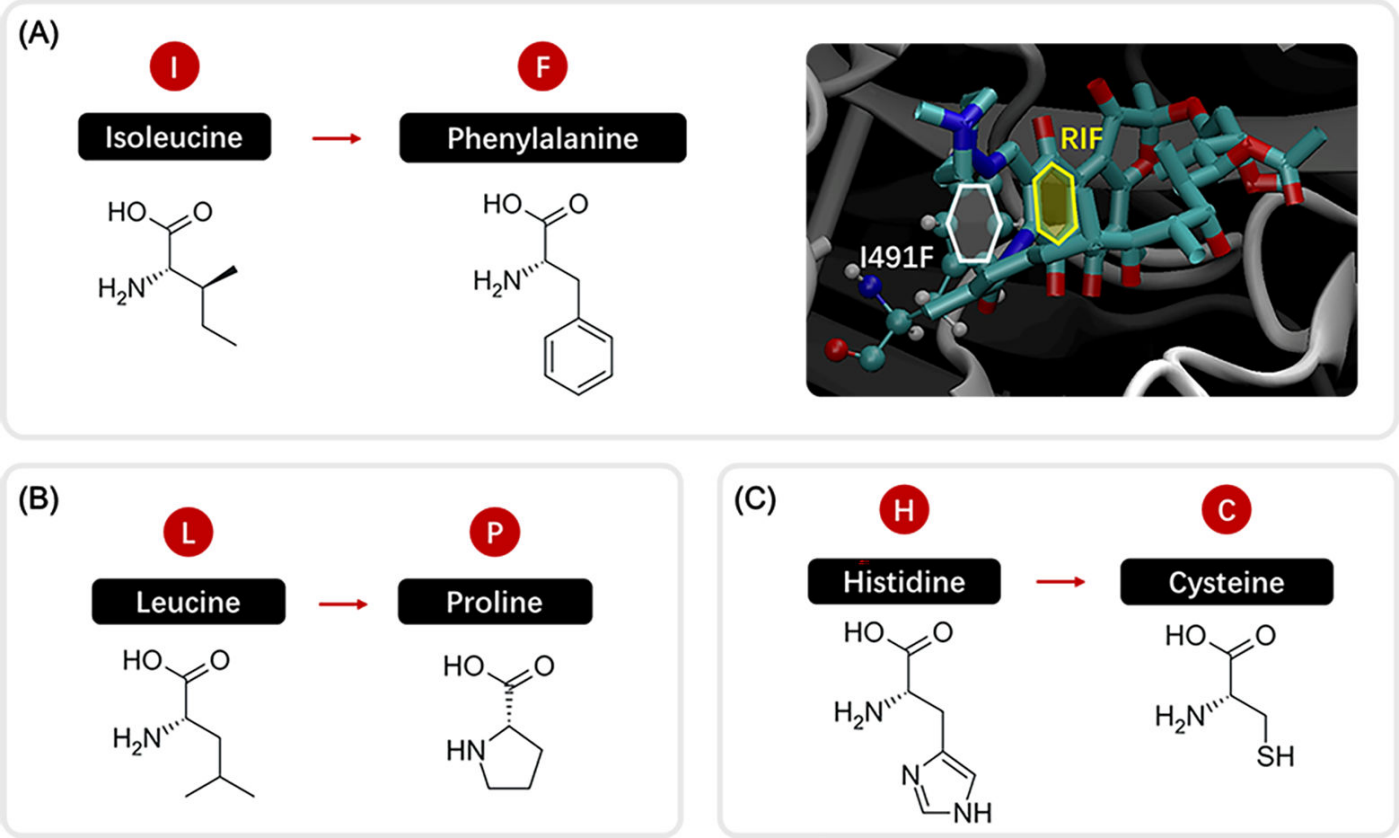
Potential factors for susceptibility predicted in three mutations. (**A**) shows the structure changes in I491F, where the benzene ring of Phenylalanine and the benzene ring in RIF end up parallel to each other. (**B**) and (**C**) show the structure changes in L452P and H445C.

The mutation I491F, which exhibits a deviation from the expected clinical phenotype, is known for its variable MIC values. Our model predicted susceptibility, conflicting with clinical data implying resistance despite a 55.5% data phenotype ratio (see Table 1). The previous study (31) also encountered difficulties with I491F, further highlighting the challenges associated with the mutation. Therefore, further analysis was conducted on the computational results. On one hand, the mutation site is located close to the binding site (3.2 Å), and the increased side-chain length provides favorable conditions for resistance. On the other hand, the mutation from isoleucine (I) to phenylalanine (F) introduces not only a longer side chain but also a benzene ring, as shown in the left part of Fig. 6A. Observation of MD simulation results revealed that this newly introduced benzene ring aligns parallel to a benzene ring within the RIF structure after energy optimization, as shown in the right part of Fig. 6A. This interaction significantly enhances the π-π stacking interaction, reducing the binding energy and thereby contributing to increased susceptibility.

Another mutation L452P shows quite similar features to I491F. From Fig. 6B, we could easily see that the structure of leucine (L) is similar to that of isoleucine (I), whereas there is also a ring in proline (P) as there is one in phenylalanine (F). With similar changes in the structures, the prediction for L452P is also in the same situation as I491F, calculated as susceptible while classified clinically as resistant, but with a clinical data of rather low phenotype ratio.

For H445C, the predicted mean ΔΔG is near zero with large uncertainty, leading to its classification as unknown. Based on Fig. 6C, the smaller size of the cysteine side chain can improve steric fit and enhance favorable van der Waals contacts in the hydrophobic pocket. It may also induce local reorganization of the conformation, stabilizing the binding site that might maintain rifampicin binding affinity and reduce the binding free energy.

To explore potential patterns in resistance mechanisms, we compared a few mutations at critical residues (D435, H445, Q432, and S450), testing 4, 8, 3, and 5 variants, respectively. As shown in Fig. 5, the regions exhibit comparable trends in mean ΔΔG and variance, which indicate that mutational position might influence the severity of resistance. These results are congruent with WHO’s conservative policy of classifying RRDR-positioned “unknown” mutations as resistant—a clinical safety-driven measure at the cost of lacking complete mechanistic support. In summary, this study indicates the robust performance of TIES_PM as an accurate computational model for rifampicin resistance prediction in *M. tuberculosis*. As a statistically robust predictor using an ensemble-based strategy with multiple replicas, TIES_PM predicts within about 5 h. TIES_PM also identified a number of mutations with inconsistent or unclear clinical phenotypes, and its molecular-level analysis unveiled possible alternative mechanisms of resistance. These results are useful for enhancing resistance mutation databases and standards of phenotype classification.

In the future, for smaller proteins, TIES_PM may be used to systematically calculate antibiotic resistance arising from all possible codon permutations, providing a comprehensive diagnostic approach. In general, it is capable of identifying and selecting leads less likely to evolve resistance during antibiotic discovery. TIES_PM provides a low-cost, fast, and scalable supplement to current diagnostic pipelines. Its platform is easily expandable to other first-line anti-tuberculosis drugs and drug-resistant pathogens, enabling personalized treatment and global resistance surveillance and control.

### Data summary

The first data set, tuberculosis_amr_catalogues repository, provides curated genetic catalogs for predicting antimicrobial resistance (AMR) in *M. tuberculosis*. These catalogs are formatted for compatibility using the Piezo Python module, enabling resistance predictions from genetic mutation data. The experimental data referenced in this study were all derived from this database: tuberculosis_amr_catalogues/catalogs/NC_000962.3/NC_000962.3_WHO-UCN-TB-2023.5_v2.0_GARC1_RFUS.csv at public · oxfordmmm/tuberculosis_amr_catalogues

The second data set is a standardized WHO catalog of mutations linked to resistance against 13 anti-TB drugs, documenting over 30,000 variants. It was established in 2021 and updated in 2023, providing essential data for genomic interpretation and molecular diagnostics.

We analyzed our calculation predictions based on the WHO-UCN-TB-2023.7-eng.xlsx from: mutation-catalogue-2023/Final Result Files at main · GTB-tbsequencing/mutation-catalogue-2023.

The third data set is generated from this study, which includes all MD simulation data. It contains input files (mutation list and a representative input template), output files (raw data and representative results), calculated ΔΔG values with errors and experimental comparisons, and Supplementary Information. Data were primarily generated on the Polaris HPC platform, supplemented by local computing resources: https://github.com/UCL-CCS/TIES_PM_RNAP_RIF.
